# Renal involvement as a unique manifestation of hemophagocytic syndrome

**DOI:** 10.3389/fmed.2022.796121

**Published:** 2022-10-05

**Authors:** Dario Roccatello, Savino Sciascia, Antonella Barreca, Carla Naretto, Mirella Alpa, Giacomo Quattrocchio, Massimo Radin, Roberta Fenoglio

**Affiliations:** ^1^University Center of Excellence on Nephrologic, Rheumatologic and Rare Diseases (ERK-net, ERN-Reconnect and RITA-ERN Member) With Nephrology and Dialysis Unit, Center of Immuno-Rheumatology and Rare Diseases (CMID), Coordinating Center of the Interregional Network for Rare Diseases of Piedmont and Aosta Valley (North-West Italy), Department of Clinical and Biological Sciences, San Giovanni Bosco Hub Hospital, University of Turin, Turin, Italy; ^2^Pathology Unit, Città della Salute e della Scienza, Turin, Italy

**Keywords:** acute kidney injury, hemophagocytic syndrome, macrophage activation syndrome, autoinflammatory diseases, hemophagocytic lymphohistiocytosis

## Abstract

Renal-limited hemophagocytic syndrome (HPS) is a rare clinical setting characterized by abnormal activation of the immune system. Fever associated with pancytopenia, hepatosplenomegaly with liver dysfunction, and hypofibrinogenemia are usually observed in HPS. From a histological level, the presence of non-malignant macrophages infiltrating bone marrow and organs represents the hallmark of this condition. Non-malignant macrophages are associated with phagocytizing activities involving other blood cells. While primary HPS is usually associated with inherited dysregulation of the immune system, secondary HPS usually occurs in the context of infection or is linked to a neoplastic process. Clinical presentation varies and can potentially lead to life-threatening settings. While renal involvement has frequently been reported, however, detailed descriptions of the kidney manifestations of HPS are lacking. More critically, the diagnosis of HPS is rarely supported by renal biopsy specimens. We report four rare cases of biopsy-proven renal-limited HPS in patients presenting with acute kidney injury (AKI). The available evidence on this topic is critically discussed in light of the possible emergence of an autonomous entity characterized by an isolated kidney involvement.

## Introduction

Hemophagocytic syndrome (HPS, additionally known as hemophagocytic lymphohistiocytosis) is a disorder characterized by an aberrant lytic response of natural killer cells (NKs) and cytotoxic CD8+ T lymphocytes (CTLs) (1–4), leading to an overactive yet less efficient immune reaction to an antigenic response. HPS can manifest as a primary (associated with an inherited dysregulation of the immune system) or acquired (usually associated with rheumatologic, infections, or oncologic processes) form. In both primary and secondary forms, the aberrant response to the target cells induces CTL proliferation, leading to an increase in interferon-γ (INF-γ) levels, thereby supporting the abnormal proliferation of benign histiocytes (macrophages). Those cells have the ability to infiltrate different tissues and organs (such as the spleen, liver, and lymph nodes). This infiltrative process is further associated with an increase of inflammatory cytokines [such as interleukin (IL)-1, IL-6, IL-18, INF-γ, tumor necrosis factor-alpha (TNF-α)] amplifying the aberrant immune response (5), leading to the *so-called cytokine storm*, clinically characterized by severe systemic inflammatory response syndrome (SIRS) and multiorgan dysfunction syndrome (MODS). Both these clinical settings are associated with a high rate of morbidity and mortality. The resulting effect is the proliferation of activated histiocytes which promotes an abnormal immune response characterized by the engulfment of circulating blood elements, such as erythrocytes, leucocytes, and platelets. Fever associated with pancytopenia, hepatosplenomegaly with liver dysfunction, and hypofibrinogenemia, is usually observed in HPS (1–4). In addition to decreased levels of circulating erythrocytes, leucocytes, and platelets, abnormal liver function tests (LFTs), hyperferritinemia, increased lactate dehydrogenase (LDH), hypertriglyceridemia, and hypofibrinogenemia are frequently seen. More recent evidence supports the presence of increased soluble CD25 levels and decreased NK cell activity. Coagulopathy can occur (4). Findings consistent with HPCs can be observed at bone marrow aspiration, albeit they are not pathognomonic for the diagnosis as these may be observed in other conditions (6, 7). In addition, infiltrates suggest that HPCs can be found in other organs, such as the spleen or liver.

The kidney can be a target organ in HPS, oftentimes clinically presenting as acute kidney injury (AKI) (4, 8). Despite a growing number of case reports confirming the potential renal involvement in HPS, evidence is still scattered and precise descriptions of the renal manifestations of HPS are still lacking. More critically, the diagnosis of HPS is rarely supported by renal biopsy specimens.

We report four rare cases of biopsy-proven renal-limited HPS in patients presenting with acute kidney injury and critically discuss the available evidence on this topic.

Clinical, laboratory, and histological evidence were collected through a chart-review analysis.

## Case 1

**Figure 1:**
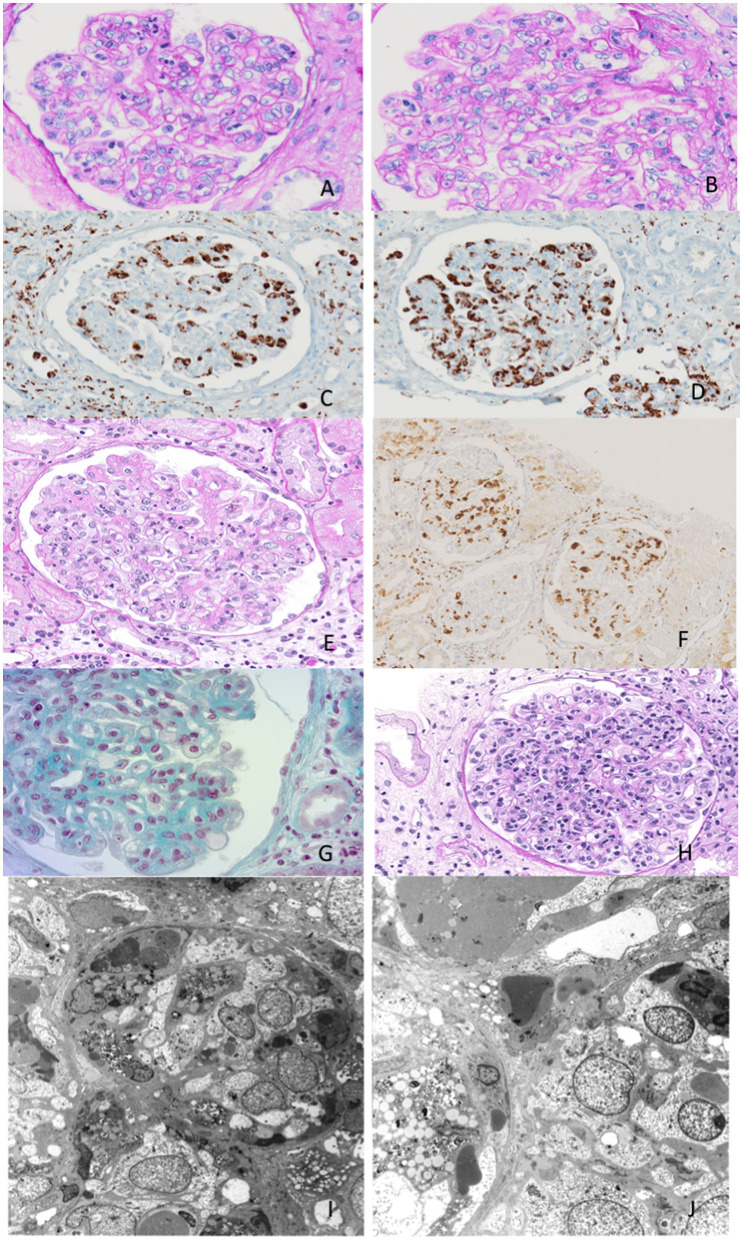
Exemplary features of renal biopsy samples from patients with hemophagocytic syndrome (HPS). **(A,B)** Diffuse thickening of the glomerular basement membrane (GBM) with double contours and capillary lumina occluded by mononuclear cells with foamy cytoplasm. **(C,D)** Immunohistochemistry shows mononuclear cells to be CD68-positive, consistent with monocyte/macrophages. **(E)** Capillary lumina occluded by vacuolized mononuclear cells. **(F)** Immunohistochemistry CD68-positive for monocyte/macrophages. **(G,H)** Capillary lumina occluded by monocyte/macrophages. **(I,J)** Features of hemophagocytosis at electron microscopy.

An 83-year-old man was transferred to our division due to a sudden worsening of renal function. At admission, he presented with confusion with spatial disorientation and anuric AKI. He had an unremarkable medical history except for alcohol abuse. He was taking antibiotics for community-acquired pneumonia associated with bilateral pleural effusion for 1 week before presentation at our center. Systemic edema was noticeable and associated with a 7-kg gain in body weight. The presence of peripheral lymphadenopathy was detectable. The main clinical and laboratory parameters are shown in Tables 1, 2. After admission, dialysis was started. Diagnostic investigations included a bone marrow biopsy whose results were unremarkable. Consequently, a kidney biopsy was performed, and histological findings are illustrated in Figure 1 and Table 3. Briefly, light microscopy showed a picture of diffuse endocapillary proliferation and focal aspects of extracapillary proliferation in the kidney with moderate vascular damage, mainly atherosclerotic. The immunofluorescence (IF) was predominantly positive for C3 (+++).

**Table 1:** Main clinical characteristics of patients with renal-limited hemophagocytic syndrome.

**Case**	**Clinical Presentation**	**Possible trigger**	**Laboratory and instrumental findings**	**Therapy**	**Outcome**
Case 1	Anuric AKI	Community-acquired pneumonia	• Bone Marrow Biopsy: unremarkable • Hb: 6.8 g/dL • WBC 1690 /μL • Plts 56 × 10^3^/μL. • sCr: 10.1 mg/dL in ESR: 71 mm/h, • CRP: 31.7 mg/dL • Ferritin: 3,520 ng/mL • C3/C4: within normal range • ANA, ANCA: negative	Steroids Pulses	Amelioration of kidney function with normalization of sCr. Resolution of the flogistic status.
Case 2	Anuric AKI	Airways infection	• Bone Marrow Biopsy: unremarkable • PET: unremarkable • CT-scan thorax and abdomen: pleural effusions and lobar pneumonia • Hb: 9.5 g/dL • WBC 2270 /μL • Plts 105 × 10^3^/μL. • sCr: 4.65 mg/dL • ESR: 45 mm/h, • CRP: 16.4 mg/dL • Ferritin: 2,500 ng/mL • C3/C4 within normal range • ANA, ANCA: negative • Bronchoscopy and Bronchoalveolar Lavage: positive for Pneumocysistis Carinii e per Herpes Simplex	Steroids Pulses, anti-viral (acyclovir) and trimethoprim-sulfamethoxazole.	Amelioration of kidney function with normalization of sCr. Resolution of the flogistic status.
Case 3	Recurrent AKI episodes	Upper airways infection	• Bone Marrow Biopsy: unremarkable • Hb: 9.7 g/dL • WBC 5,930/μL • Plts 153 × 10^3^/μL. • Proteinuria: 5 g/25 h • sCr: 3.7 mg/dL in • ESR: 63 mm/h, • CRP: 13,2 mg/dL	Canakinumab	Stabilization of kidney function. No further episode of AKI
Case 4	Deterioration of renal function in patient in F/U for previous renal carcinoma	Community-acquired pneumonia	• Bone Marrow Biopsy: unremarkable • PET: unremarkable • Hb: 8.5 g/dL • WBC 2,190 /μL • Plts 96 × 10^3^/μL. • sCr: 2.0 mg/dL • ESR: 41 mm/h, • Proteinuria: 2.5 g/25 h • CRP: 18.5 mg/dL • Ferritin: 284 ng/mL • C3: within normal range • C4: 5 mg/dl • Total cholesterol: 249 mg/dl • ANA, ANCA: negative	Anakinra	Stabilization of kidney function.

**Table 2:** Features suggestive of reactive hemophagocytic syndrome included in the HScore.

**Case**	**HScore items#**
Case 1	3 lineages of cytopenias and elevated ferritin
Case 3	3 lineages of cytopenias and elevated ferritin
Case 3	1 lineage cytopenia, high temperature
Case 4	3 lineages of cytopenias and elevated ferritin

According to # Fardet et al. (23).

**Table 3:** Histological findings at kidney biopsy.

**Patient**	***n*. glomeruli**	**IF**	**Histological description**	**IFTA**	**EM**	**Findings in line with HPS**
#1	21	C3+	Diffuse endocapillary proliferation and focal aspects of extracapillary proliferation in a kidney with moderate vascular damage, mainly atherosclerotic.	-	Presence of histiocytes and a characteristic feature of hemophagocytosis	CD68 positive cells were observed at immunohistochemistry; feature of hemophagocytosis at EM
#2	28	C3+	Focal segmental glomerulosclerosis with areas of interstitial fibrosis with tubular atrophy. Capillary lumina were occluded by vacuolized mononuclear cells.	Areas of interstitial fibrosis with tubular atrophy.	Presence of histiocytes and a characteristic feature of hemophagocytosis	CD68 positive cells were observed at immunohistochemistry; feature of hemophagocytosis at EM
#3	14	-	Capillary lumina were occluded by vacuolized mononuclear cells, giving rise to foamy-appearing glomeruli.	-	Presence of histiocytes and a characteristic feature of hemophagocytosis	CD68 positive cells were observed at immunohistochemistry; feature of hemophagocytosis at EM
#4	13	-	Capillary lumina occluded by vacuolized mononuclear cells, giving rise to foamy-appearing glomeruli (PAS++). Mild thickening of the basal membrane.	Diffuse interstitial fibrosis with tubular atrophy.	Feature of hemophagocytosis	CD68 positive cells were observed at immunohistochemistry; feature of hemophagocytosis at EM

These features were compatible with glomerulonephritis with dominant deposits of C3. However, numerous CD68-positive cells were observed at immunohistochemistry, while electron microscopy examination confirmed the presence of histiocytes and a characteristic feature of hemophagocytosis. These findings were strongly evocative of HPS.

However, it is worth mentioning that, despite the histological features, our case did not fully meet the current classification criteria for HPS.

## Case 2

A 79-year-old man was transferred to our division because of a severe worsening of his kidney. He presented with AKI with a serum creatinine (sCr) level of 4.65 mg/dl, hyperazotemia (487 mg/dl), thrombocytopenia (platelet count, 10.5 × 10^4^/μl), and ferritin level was 2,500 ng/ml. The blood pressure was 80/50 mm Hg. Furthermore, in this case, the bone marrow biopsy was unremarkable (Tables 1, 2).

To determine the severity of the glomerulopathy of the patient, a kidney biopsy was performed (Figure 1, Table 3).

## Case 3

A 69-year-old man was admitted for evaluation of recurrent AKI episodes of unknown origin. His previous medical history was characterized by acute coronary events and chronic obstructive pulmonary disease. AKI episodes were referred to as being anticipated by massive proteinuria (up to 20–25 g/day). At the first evaluation at our center, he presented with a new onset of AKI and fever (38.2°C) 10 days after an episode of upper airway infection. The main clinical and laboratory parameters are shown in Table 1. A kidney biopsy was performed (Figure 1). Immunohistochemistry showed mononuclear cells to be CD68-positive, consistent with monocyte/macrophages. Capillary lumina are occluded by vacuolized mononuclear cells.

## Case 4

A 70-year-old woman was admitted for evaluation because of the onset of fever (38.2°C), edema in the lower extremities, and deterioration of renal function (sCr 2 mg/dl). Laboratory findings showed non-nephrotic range proteinuria (2.5 g/24 h) (Table 1). Her previous medical history showed MGUS and well-controlled arterial hypertension. Nephrectomy due to renal cell carcinoma was conducted 10 years before admission, leading to a moderate loss in renal function (sCr 1.4 mg/dl). With the deteriorating conditions of the patient, a kidney biopsy was carried out. Immunohistochemistry shows that CD68 is present in monocyte/macrophages.

## Renal involvement in HPS

A spectrum of clinical presentations and histological findings has been associated with renal involvement in HPS over the years.

### Clinical features

Acute kidney injury is the most frequently reported renal complication in HPS. Out of 95 patients with secondary HPS admitted to the intensive care unit (ICU), Aulagnon et al. (9) found a rate of AKI as high as 62%, with most of them in stage 2 or 3 AKI. Dialysis was initiated in almost 60% of the included patients. Hypoperfusion and acute tubular necrosis were identified as the main causes of AKI, albeit tumor lysis and glomerulopathy were similarly described in the cohort. However, histological renal confirmation was obtained in only 1 case. About 12% of the cohort had nephrotic syndrome (NS) which was associated with AKI in two-thirds of the cases. Among the survivors, 23% of patients had CKD when evaluated 6 months after the diagnosis of HPS. A recent retrospective study by Kapoor et al. (10), when analyzing the intensive care unit complications and outcomes of adult patients with HPS, showed that septic shock was the most common ICU complication seen in 14 (88%) patients, followed by AKI (81%) with the majority of the patients requiring renal replacement therapy.

In addition, when analyzing available literature, it is worth noting that most of the reported cases of HPS with renal involvement are described in the context of renal transplant (11–13).

Risdall et al. (14) reported a series of 19 subjects (68% renal transplant recipients) in whom HPS was triggered by infection, mainly cytomegalovirus (CMV). In a larger cohort analysis, Karras et al. (15) found a prevalence of 0.4% for HPS among 4,230 renal transplant recipients when investigating 17 HPS subjects (82% of those with confirmed viral infection). HPS occurred a median of 52 days after transplantation. Asci et al. (16) identified 13 patients with HPS out of more than 400 renal transplant recipients (a prevalence of 3.2%). HPS was observed for an average of 15 months after transplantation. In line with the other reports, an infectious trigger was found in the majority of the cases. Notably, HCV infection was reported in more than 50% of the cases. A larger cohort including more than 70 cases of HPS identified among kidney transplant recipients was described by Ponticelli et al. (17). While in most of the subjects, HPS was triggered by viral infections, bacterial and protozoal infections were also described. The observed overall mortality rate was above 50%. Since these first descriptions, a constellation of further cases of HPS associated with different infective diseases has been described in patients with kidney transplant (18–20).

Very recently, Wang et al. (21) analyzed 600 patients with adult secondary HPS attempting to identify risk factors associated with AKI occurrence in this setting. They found that several clinical (heart failure, gastrointestinal symptoms, disseminated intravascular coagulation, high heart rate at admission, and need for vasopressors) and laboratory parameters (increased serum phosphorus, total bilirubin, and low albumin levels) were independently associated with an increased risk of developing AKI. Similarly, they observed that the administration of vasopressors, AKI stage III, baseline Cystatin-C, total bilirubin, number of days of glucocorticoid therapy, fibrinogen level, and the presence of multi-organ failure were found to be independent risk factors for in-hospital mortality (21).

### Renal histological findings in HPS

While the abovementioned studies provided clinical insights to improve the understanding of renal involvement in HPS, they only marginally contributed to the fine characterization of histological injuries.

First, it is worth mentioning that the clinical entity hemophagocytic lymphohystiocytosis should not be confused with the related histological findings (hemophagocytosis).

Second, from a pathogenic perspective, AKI in HPS seems to be supported by the interstitial infiltration of CTLs and activated macrophages (12).

Acute kidney injury in HPS results from inflammatory or ischemic lesions of the renal tubules. Acute tubular necrosis is the most frequent feature of renal damage in HPS. Fitzgerald et al. (22) reported the prevalence of this finding in up to 45% of patients with HPS in an autopsy series. In more than half of these cases, the presence of acute tubular necrosis was associated with interstitial inflammation (22). Tubulointerstitial lesions might be related to both hemodynamic alteration or coagulation disorders (e.g., disseminated intravascular coagulopathy) conditions that can occur during the acute phase of severe sepsis.

Glomerular involvement is uncommon and usually manifests as either podocytopathy with collapsing glomerulopathy or thrombotic microangiopathy. In our cohort, two patients presented with proteinuria, one in the nephrotic range and one in the sub-nephrotic range. The injuries leading to NS and glomerular damage are not fully defined and seem to be associable with primary podocyte involvement. Thaunat et al. (12) described a cohort of nine biopsy-proven patients with HPS presenting with NS and compared their experience with available cases in the literature. Histological patterns of renal injuries associated with HPS were as follows: collapsing focal segmental glomerulosclerosis (mainly seen in subjects of African descent), minimal change disease [all in White subjects, as also described in a previous report (13)], and thrombotic microangiopathies features seen in the remaining two cases.

More recently, a case of HPS with histiocytic glomerulopathy and intraglomerular hemophagocytosis has also been reported. Histological features showed massive glomerular infiltration by macrophages resembling proliferative glomerulonephritis accompanied by intraglomerular hemophagocytosis and mild features of glomerular thrombotic microangiopathy (24).

### Therapeutic options

To date, the management of renal limited HPS has been based on anecdotal experience.

The grade of hyperinflammation, triggering condition, and eventual concomitant diseases are among the factors to consider when choosing the therapeutic options for renal involvement in HPS. Similarly, the presence of underlying genetic abnormalities should be investigated when possible. To date, no therapy has been trialed in a randomized fashion for this condition, and available experience mainly relies on observational studies or anecdotal case reports. Inherited forms range in severity, with familial hemophagocytic lymphohistiocytosis being associated with < 5% survival rate at 12 months (19). The so-called HPS-94 protocol (based on the Histiocyte Society initiated the prospective international collaborative therapeutic study, launched in 1994) includes the use of 8 weeks of dexamethasone and etoposide with intrathecal methotrexate and has been associated with positive outcomes in selected cases. In some subjects with familial or relapsing forms, a scheme with daily cyclosporine associated with dexamethasone pulses and intermittent etoposide was also considered (24). This approach was shown to improve the estimated 5-year survival by up to 54% in a multinational study including more than 240 pediatric patients. Survival rates reached 66% in the 124 patients who underwent hematopoietic stem cell transplantation (24). In total, 49 subjects were alive after 12 months of observation time after the therapy, posing the question of whether the observed heterogeneity in the outcomes could have also been explained by a certain prevalence of secondary HPS included in the study cohort. Variations to the HPS-94 scheme have been proposed over the years, including the HPS-2004 protocol (adding cyclosporine during the 8-week induction phase) (25). The addition of anti-thymocyte globulin (ATG) has also been described, with an observed improvement in the complete response rate. The use of intermittent intravenous immunoglobulin or cyclosporine has been proposed as a maintenance therapy (26). Hematopoietic stem cell transplantation is usually considered in the most severe cases (familial, relapsing, or refractory forms) or in cases of documented genetic mutations. Reduced-intensity conditioning seemed to have a better tolerability profile compared with myeloablative conditioning in this setting.

As in primary HPS, the treatment of secondary HPS in adults is still under debate. The main challenge in the management of this condition is to control the hyperinflammatory state. However, one should also acknowledge that anecdotal experiences report positive outcomes with only supportive therapy and adequate treatment of the trigger. Among others, it seems that the use of amphotericin as an antimicrobial agent may be sufficient for leishmania-triggered HPS.

High-dose corticosteroids are usually suggested for patients requiring urgent care. The HPS-2004 scheme should be considered in the most severe cases (familial or relapsing forms). Due to its selective activity on CD8 + CTLs as proven in lymphocytic choriomeningitis virus-infected Prf–/–mice, etoposide can be considered a logical candidate agent for HPS (27). Similar effects have also been described for methotrexate and cyclophosphamide. A significant improvement in 1-month survival was associated with the first-line use of etoposide after multivariable analysis in a retrospective study involving 162 adults with sHPS (28). The management of the hyperinflammatory state should be paralleled by the identification of the eventual infectious trigger and consequent therapy should be arranged. In cases triggered by EBV, observational evidence supports the efficacy of early use of etoposide (within 1 month from symptoms onset) in the management of EBV-triggered HPS, in both pediatric (29) and adult (30) populations. The rationale for the use of etoposide relies on data demonstrating EBV infection of CD8 + CTLs in EBV-triggered HPS (31, 32). Additionally, direct antiviral effects have been speculated *in vitro* for etoposide due to its ability to inhibit EBNA synthesis and reduce the EBV-induced transformation of mononuclear cells (33). Hematopoietic stem cell transplantation has been proposed for the management of EBV-HPS (34). As B-lymphocytes might also play a role in EBV-triggered HPS (35), the use of Rituximab as add-on therapy has been investigated, showing the ability to reduce ferritin levels and EBV titers (36).

Some additional considerations are worth mentioning when discussing hyperferritinemia status in the context of multiple organ dysfunction syndromes, a condition occurring in a not negligible proportion of patients in specific settings, such as intensive care units. Malignancies, active rheumatologic conditions, coagulopathies, or trauma have been identified as possible triggers for HPS in addition to infections (37, 38). When managing the trigger event, the presence/suspicion of a septic status poses some challenges for the use of chemo-immunotherapy. Conversely, a family history of HPS or evidence of genetic abnormalities obtained by a rapid flow cytometric screening could support the use of intensified schemes, such as the HPS-2004 protocol (39). Two recent series describe mortality and treatment of the adult HPS cases admitted to the ICU, one based on HPS-2004 criteria (37) and one based on the HScore (38). According to two recent studies, ICU hospital mortality rates ranged from 52 to 68% (37, 38). The studies differ based on the criteria used [HPS-2004 criteria (37) or HScore (38)], steroids administration rate (55 vs. 66%), etoposide (80 vs. 40%), and intravenous immunoglobulin (5 vs. 27%) use. In addition, in the ICU settings, some authors preferred methylprednisolone over dexamethasone (40). The use of plasma exchange (PE), with or without additional therapies, including biological agents has also been investigated. In total, 23 pediatric patients with hyperferritinemia suspected of sHPS identified in a retrospective analysis were treated with PE and either IVIG or methylprednisolone. Outcomes were compared with PE and IVIG with dexamethasone, cyclosporine, or etoposide. Although a high rate of infections was observed, only three patients died, all were among those who received the HPS-204 like regimen (40). The combination of PE and IVIG has been further supported in other series (41, 42).

As previously mentioned, active malignancy, especially lymphomas, can act as a trigger for HPS. Indeed, HPS can be directly associated with the active phase of the oncologic condition or can develop as a result of immunosuppression, where it is usually triggered by an infection (4, 43). In some cases, the two conditions can co-exist with EBV being the most frequently reported infection in this setting (4). In such cases, anti-B cell therapy may be recommended in addition (43). With active neoplasm, it remains uncertain whether the first-line management should be primarily HPS-targeted (e.g., HPS-2004 scheme) or if the priority should be given to targeting the specific malignancy. Up to half of the cases of HIV-related HPS cases are associated with active malignancy, mainly lymphoma (44).

Stopping (or at least reducing) the ongoing chemotherapy should be pondered in the case of chemotherapy-related HPS resulting from infection (43).

When compared with familial hemophagocytic lymphohistiocytosis or other forms of HPS, the prognosis seems more benign in cases triggered by autoinflammatory/autoimmune conditions. Among others, for example, the observed mortality rate was < 10% in the HPS cohort of patients with systemic juvenile idiopathic arthritis (45). In these settings, initial management is usually less aggressive than in the HPS-2004 scheme and avoids the use of etoposide. In the previously mentioned study including patients with systemic juvenile idiopathic arthritis (45), nearly all patients were given steroids, more than half received cyclosporine, while a third was managed with IVIG. Similar therapeutic approaches have been reported in a series of 39 juvenile lupus-associated HPS, with an overall reported mortality of approximately 10% (46). These figures are also mirrored in one of the largest series available including 116 adult patients with HPS-related autoimmune/autoinflammatory conditions in whom the coexisting of active infection or malignancy was excluded. With an overall mortality rate below 15%, the use of steroids seemed to be associated with a favorable prognosis, even when given in monotherapy (about half of the cases) (4, 47). Steroids were often associated with IVIG, cyclosporine, or, less frequently, IV cyclophosphamide. Only a strict minority of the cases were exposed to etoposide.

The management of HPS in kidney transplant recipients is based on anecdotal reports. Available data support the concept that the majority of cases are usually associated with infections, making the use of antimicrobial therapy a priority. Calcineurin inhibitor therapy could not be discounted, also taking into account the data coming from studies investigating the efficacy and safety of the HPS-2004 protocol. High-dose steroids are often preferred, while the concomitant use of anti-metabolite agents is discouraged to avoid over-immunosuppression. IVIG could be considered in the case of rejection, with the use of PE to be preferred if antibody-mediated. The combined use of high-dose steroids, etoposide, and rituximab seems reasonable in the case of EBV infection.

With the current increase in the options available for the management of autoimmune conditions, biologic agents have been applied in the treatment of HPS, with anakinra the most commonly used. Anakinra was initially proposed as a promising effective option for the management of sHPS in a retrospective case series of eight critically ill pediatric patients (46). While the therapy was well tolerated, one should also acknowledge that patients were also receiving high-dose steroids and IVIG. On the other hand, its use is safe in patients with severe sepsis (47). Other biological agents, such as anti-TNFα drugs and anti-IL-6 (tocilizumab), have been used for rheumatoid arthritis and adult Still's induced HPS] (4). More recently, canakinumab has been proposed in selected refractory cases of Still's induced HPS (48). Notably, HPS can also occur in patients with inflammatory arthritis receiving biological agents, such as tocilizumab (4, 49, 50), canakinumab (51), and anakinra (52). Anakinra dose escalation has been reported in such cases. B-cell target therapies (e.g., rituximab, or more recently, belimumab) have been proposed for SLE, and EBV-associated HPS with or without associated lymphoma (4).

### Renal involvement as a unique manifestation of hemophagocytic syndrome

To date, there is no unanimous agreement on the nomenclature for HLH- and MAS-related diseases (53). While we acknowledge that the reported cases might not fully match the classification proposed for HPS, the clinical onset, the laboratory profiles, and the histological findings (albeit not pathognomonic) might be in line with a form of acquired HLH with the kidney as the main organ involvement. In detail, our patients meet some of the HLH-2004 criteria (Tables 1, 2) (54). However, the clinical applicability of those criteria is limited as they include some specialized diagnostic tests, such as soluble IL-2 receptor alpha levels (sCD25), NK-cell functional assays, and genetic testing that might not be routinely available. More critically, some of the HLH-2004 items are not pathognomonic for HLH either. For instance, hemophagocytosis is not always found on bone marrow biopsy in patients with HLH, particularly in the early phase of the disease (54–56).

When referring to the HScore (23), a score of ≥250 has been associated with a high probability of acquired HLH (>99%), however, its use has never been investigated in forms of acquired HLH with mainly organ-limited manifestations. Our pilot observation might support the concept that in selected cases presenting with severe renal involvement and some signs/symptoms in line with HLH (once excluded concomitant possible differential diagnoses), a form of renal involvement as a unique manifestation of the hemophagocytic syndrome could be considered, especially when histological evidence of CD68 histiocytes is present.

One might speculate that our cases could represent a subgroup of secondary HLH or, when specifically referring to cases #1, #2, and #4, a novel hyperferritinemia syndrome identifiable in adults with a florid inflammation, increased ferritin, and AKI with macrophages at kidney biopsy. All in all, our report describes a potentially new condition (which shares some links with HLH) with renal involvement characterized by extensive activated macrophage infiltration of the glomeruli and moderate signs of systemic inflammation. This condition can be identified only by a renal biopsy.

## Conclusion

The main aim of our paper was to increase the awareness of HPS as a potential cause of AKI, with or without multiorgan failure, among nephrologists. While renal involvement has frequently been reported in patients with overt cases of HPS, one should bear in mind that occasionally HPS cases can be diagnosed solely based on the findings of renal biopsy. When AKI can develop in the context of critically ill patients (9), distinguishing HPS from severe SIRS/MODS secondary to sepsis, trauma, or autoimmune/autoinflammatory diseases can be challenging. However, renal histological discrimination is crucial, as immunomodulatory therapy may be needed to control the hyperinflammatory state once HPS has occurred, but may be harmful otherwise. However, clinicians should be aware that the availability of electron microscopy is requested to identify such cases.

## Data availability statement

The raw data supporting the conclusions of this article will be made available by the authors, upon reasonable request.

## Ethics statement

Ethical review and approval was not required for the study on human participants in accordance with the local legislation and institutional requirements. The patients/participants provided their written informed consent to participate in this study.

## Author contributions

All authors listed have made a substantial, direct, and intellectual contribution to the work and approved it for publication.

## Conflict of interest

The authors declare that the research was conducted in the absence of any commercial or financial relationships that could be construed as a potential conflict of interest.

## Publisher's note

All claims expressed in this article are solely those of the authors and do not necessarily represent those of their affiliated organizations, or those of the publisher, the editors and the reviewers. Any product that may be evaluated in this article, or claim that may be made by its manufacturer, is not guaranteed or endorsed by the publisher.
